# Mouse Models of Gastric Cancer

**DOI:** 10.3390/cancers5010092

**Published:** 2013-01-24

**Authors:** Yoku Hayakawa, James G. Fox, Tamas Gonda, Daniel L. Worthley, Sureshkumar Muthupalani, Timothy C. Wang

**Affiliations:** 1 Department of Medicine and Irving Cancer Research Center, Columbia University Medical Center, New York, NY 10032, USA; 2 Division of Comparative Medicine, MIT, Cambridge, MA 02139, USA

**Keywords:** gastric cancer, mouse model, metaplasia, *Helicobacter felis*, *Helicobacter pylori*, INS-GAS mice

## Abstract

Animal models have greatly enriched our understanding of the molecular mechanisms of numerous types of cancers. Gastric cancer is one of the most common cancers worldwide, with a poor prognosis and high incidence of drug-resistance. However, most inbred strains of mice have proven resistant to gastric carcinogenesis. To establish useful models which mimic human gastric cancer phenotypes, investigators have utilized animals infected with *Helicobacter* species and treated with carcinogens. In addition, by exploiting genetic engineering, a variety of transgenic and knockout mouse models of gastric cancer have emerged, such as INS-GAS mice and TFF1 knockout mice. Investigators have used the combination of carcinogens and gene alteration to accelerate gastric cancer development, but rarely do mouse models show an aggressive and metastatic gastric cancer phenotype that could be relevant to preclinical studies, which may require more specific targeting of gastric progenitor cells. Here, we review current gastric carcinogenesis mouse models and provide our future perspectives on this field.

## 1. Introduction

Gastric cancer remains the 2nd leading cause of cancer mortality worldwide, with an overall 5-year survival rate that is less than 25% [1,2]. Critical to understanding the mechanisms involved in gastric cancer, and devising preventive and therapeutic interventions, is the need to develop an authentic animal model. Mice have a different gastric anatomy compared to humans. In mice the gastric fundus equivalent is lined by squamous, rather than oxyntic glandular epithelium. Thus, in mice, the squamocolumnar junction does not universally approximate the gastroesophageal junction as it does in normal human anatomy. In addition, rodents rarely develop spontaneous gastric cancer, although some reports have described spontaneous gastric adenocarcinomas in cotton rats (*Sigmodon hispidus*), and in the Z strain of the African rodent *Mastomys natalensis* [3,4,5,6,7]. However, these animals when they develop gastric tumors more frequently exhibit enterochromaffin-like cell carcinoids. Thus, experimental efforts have focused on identifying chemical, infectious or genetic means to induce gastric cancer in animals.

Prior to the discovery of *Helicobacter pylori* infection, major etiological factors were thought to be a diet rich in salt and nitrates/nitrites, along with a low intake of ascorbic acid and carotenoids [8], and thus early animal models utilized nitrosamines, such MNNG in rats [9,10] and MNU in mice to induce gastric tumors [11].

With the discovery of *H. pylori* by Marshall and Warren [12], and subsequently studies showing a strong association with gastric cancer [13,14,15,16,17], the focus shifted to the development of animal models of *Helicobacter*-associated gastric cancer. A variety of animals, including mice, rats, Mongolian gerbils, cats, guinea, pigs, ferrets, pigs, and macaques, have been experimentally infected with a variety of different *Helicobacter* species. Because of the ability to manipulate the mouse genome, mice have become the animal model of choice for cancer research. While the greatest interest has been in mouse models, only a limited number of *H. pylori* strains have been identified that successfully colonize the mouse stomach. The most robust and useful models to date have been *H. pylori* SS1-infected mice, and *H. felis* originally isolated from the stomach of cats and dogs [18]. Both of these gastric *Helicobacters* are capable of long-term colonization and have the ability to induce chronic gastritis and precancerous lesions in mice. However, the SS1 strain is not able to induce gastric cancer in most inbred strains of mice, while it does cause gastric carcinoma in C57BL/129 mice [19,20]. Chronic *H. felis* infection has been shown to induce severe inflammation, atrophy, metaplasia, dysplasia and gastric cancer in C57BL/6 mice [21].

While genetically engineered mouse models of cancer were developed in the 1980’s, transgenic models of gastric cancer were slow to emerge. Initial models included some containing a variety of oncogenes that were known to transform but had no known association with human gastric cancer, such as the SV40 T antigen, which binds to pRb and disrupts its function [22]. Human carcino-embryonic antigen (CEA) promoter/SV40 T antigen transgenic mice were reported to develop antral hyperplasia or gastric cancer [23,24]. Stomach-specific SV40 T antigen transgenic mice using H/K-ATPase-β subunit gene promoter [25] developed hyperplasia and abnormal cell distribution within the gastric glandular unit, and rarely developed dysplasia. Other early models included transgenic mice carrying the human adenovirus type 12 (Ad12) early region 1 under control of the mouse mammary tumor virus (MMTV) long terminal repeat (LTR), which developed adenocarcinoma or adenosquamous carcinoma [26]. In addition, transgenic mice expressing HPV-16 early region of the bovine keratin 6 gene promoter developed glandular stomach tumors [27]. However, these models did not progress to cancer through the atrophy-metaplasia-dysplasia sequence, as described by Correa [28], nor were they associated with *Helicobacter* infection or chronic inflammation.

INS-GAS mice as a model of spontaneous gastric cancer were first described in 2000. These mice were reported to develop atrophic gastritis and intestinal metaplasia, followed by corpus cancer with a high incidence rate, and tumor development was accelerated by *H. felis* infection, suggesting that this model closely mimicked the clinical course of human gastric carcinogenesis [29]. This initial mouse model was followed by the H/K-ATPase-IL-1β transgenic mice, which progressed through the atrophy-metaplasia-dysplasia sequence and validated human genetic data that implicated the IL-1β gene locus as a major risk factor for gastric cancer [30]. Other studies using genetic mouse models of gastric cancer have been reported, and are detailed below. These genetic mouse models have provided considerable insight on the role of the stroma, also known as the tumor microenvironment, which has been recognized as a critical factor in various types of cancer. Modulation of cytokines, chemokines or the signaling pathways upstream has also demonstrated unequivocally the importance of inflammatory responses in gastric cancer development [31,32,33,34].

Nevertheless, despite the significant advances made utilizing diverse mouse models, these models have all shown some limitations, including modest gastric pathology, slow time course, and the absence of invasive or metastatic tumors. In addition, it is important to keep in mind that the response to infection or genetic manipulation is highly dependent on the mouse genetic background, gender, diet and housing conditions. Animal stress associated with overcrowding, inadequate sanitation, and variations in temperature, humidity, and light cycles may predispose resistant animals to adverse disease outcomes. Especially in the case of the enterohepatic *Helicobacter* species, differences in study outcomes may be attributed to persistent colonization by these murine *Helicobacters* [35,36,37].

Here we review these genetic or chemical models of gastric carcinogenesis, and compare their pathological features, limitations and contribution to our understanding of gastric carcinogenesis. We also emphasize the impact of comprehensive genomic analysis on new or emerging transgenic mouse models.

## 2. Chemical Carcinogenesis Models of Gastric Cancer

To explore the mechanisms of gastric cancer development and establish a useful animal model of gastric tumorigenesis, investigators examined the utility of a variety of chemical carcinogens. In particular, researchers focused on *N*-nitroso compounds, which are generated in the stomach by anaerobic bacteria following ingestion of nitrates and nitrites, which were thought to be an important inducer of human cancer. *N*-methyl-*N*-nitro-*N*-nitrosoguanidine (MNNG) was the first nitrosamine shown by researchers to induce stomach tumors in rats. In 1966, Schoental *et al.* administered MNNG to rats using a stomach tube, resulting in squamous cell carcinoma in the rat forestomach [38]. In 1967, Sugimura *et al.* modified their method, administering MNNG orally to rats continuously in the drinking water, and achieved for the first time a high incidence of antropyloric adenocarcinoma [10]. MNNG was later found to be a very potent gastric carcinogen in Mongolian gerbils [39,40]. Exposure to 400 ppm MNNG in drinking water for 50 weeks resulted in gastric adenocarcinomas in 63.6% of gerbils [40]. Using MNNG-induced gastric cancer model, it has been reported that administration of high-salt diet [41,42], calcium-deficient diet [43], catechol [44], or IL-1β [45] promotes gastric cancer development.

Rats and gerbils are limited as model systems, given the absence of genetic models, and thus investigators explored the effect of oral administration of nitrosamines in inbred strains of mice. However, mice proved to be remarkably resistant to MNNG-induced gastric carcinogenesis. Danon *et al.* infected female Balb/c mice with *H. heilmannii* and administered 150 ppm MNNG in drinking water for 38 weeks and found that the treated mice developed squamous cell carcinomas in the mouth and forestomach, but not adenocarcinoma in the glandular stomach [46].

**Figure 1:**
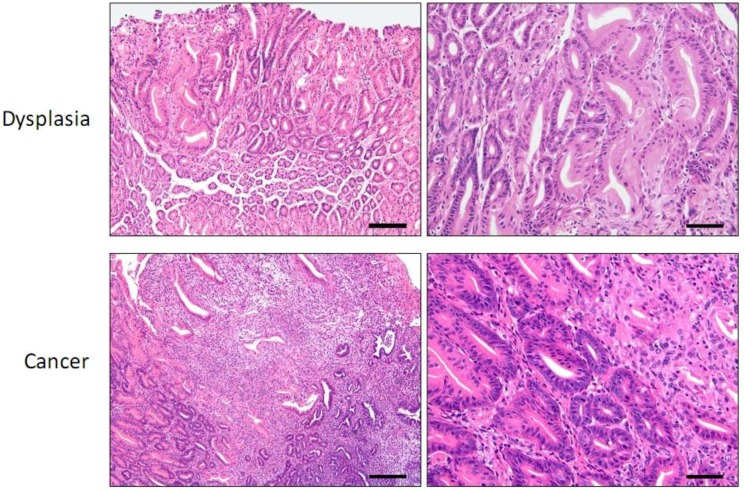
MNU-induced mouse tumor. Top panels: Low and high magnification images of the distal corpus showing gastric dysplasia (glandular proliferation with architectural distortion and cytological atypia in the superficial half of the mucosa) associated with oxyntic loss and pyloric-type glandular metaplasia. (Bar: Left 200× or 80 μM, Right 400× or 40 μM) Bottom panels: Low and high magnification images of a gastric tumor biopsy specimen showing dysplastic glands with lamina propria invasion, effacement and associated desmoplasia and inflammation. (Bar: Left 100× or 160 μM, Right 400× or 40 μM).

Researchers then explored the utility of *N*-methyl-*N*-nitrosourea (MNU) as a gastric carcinogen in mouse models. Tatematsu *et al.* treated Balb/c mice with 0.5 mg MNU by weekly intragastric intubation, but found that most of the mice died due to squamous cell carcinoma in the forestomach. Interestingly, when the mouse forestomach was removed surgically prior to MNU treatment, well-differentiated adenocarcinoma developed in the glandular stomach with 100% incidence rate by 40 weeks [11]. Thus, the glandular stomach was indeed sensitive to the carcinogenic effects of MNU, but the phenotype was obscured by the greater sensitivity of the forestomach, at least at that specific dose and route of administration. Tatematsu *et al.* went on to demonstrate that 30–120 ppm MNU given in drinking water was preferable to oral gavage, without the induction of tumors of the forestomach [47]. The efficiency of tumor induction by MNU was found to depend on its concentration rather than total intake [48], and MNU in the drinking water at 240 ppm on alternate weeks (total exposure; 5 weeks) was effective in inducing gastric cancer in 6 strains of mice that were studied [49]. Consequently, the protocol of 240 ppm MNU treatment in the drinking water for 5 weeks (every other week) is currently a standard and widely used murine method of gastric carcinogenesis. MNU-induced tumors in mice are located mainly in the gastric antrum, and pathologically are uniformly well- or moderately-differentiated adenocarcinomas (Figure 1). The tumors are rich in stromal cells, and occasionally invade into the submucosa although signet ring-cell carcinoma and metastatic tumors are rarely, if ever seen.

The MNU mouse model of gastric cancer has been extensively used for investigating the role of various signaling pathways or transcription factors in gastric carcinogenesis, including the roles of p53 [50], NF-κB [51], MAPK pathway [52,53], Cox-2 [54,55], β-catenin [54], E-cadherin [56], and KLF4 [57]. Although MNU is an alkylating agent that can potentially induce the formation of DNA adducts and GC > TA transversion mutations, only rare mutations have been observed in *N*-nitroso-compounds (NOC)-induced gastric tumors of rodents [58], raising questions about the precise mechanism of carcinogenesis. MNU is also known to modify amino acids in histone proteins, especially histone H3 lysine residues, leading to chromatin remodeling [59]. Indeed, MNU treatment in mice affects the expression of TFF1, an important gastric-specific tumor suppressor gene, through epigenetic modifications. DNA and histone methylation, and promoter methylation of the TFF1 gene have also been observed in human gastric cancer [60]. This suggests that epigenetic effects are likely to constitute a key mechanism of NOC-induced carcinogenesis.

One potential criticism of the MNU mouse model of gastric cancer is the absence of *Helicobacter* spp.-associated chronic inflammation. While the MNU model does not proceed through a classical atrophy-metaplasia-dysplasia sequence, this latter *H. pylori*-dependent pathway results in achlorhydria with subsequent bacterial overgrowth; NOCs may be generated from nitrates and nitrites in this setting, and thus the argument can be made that the generation of *N*-nitroso compounds may play a role in *Helicobacter*-associated carcinogenesis. A recent study used a combination of MNU and *H. felis* infection [60], and achieved a very rapid induction of antral gastric cancer. This same combination was reported to induce a high frequency of gastric cancer in *H. pylori-*infected Mongolian gerbils compared to gerbils receiving MNU only [61,62]. Thus, the combination of *Helicobacter* spp. infection followed by MNU treatment mimics in some ways the proposed pathogenesis of human antral carcinogenesis.

## 3. *Helicobacter* Infection Models

Given the key role of *Helicobacter pylori* infection in the etiology and pathogenesis of gastric cancer, researchers pursued the development of animal models of gastric *Helicobacter* infection. The first studies supporting a potent carcinogenic role for *Helicobacter* species in the gastric mucosa was the ferret model of *Helicobacter mustelae* infection [63,64,65]. Ferrets naturally infected with *H. mustelae* exposed to one dose of 100 mg/kg MNNG developed gastric cancer, while *H. mustalae* infected ferrets did not [63]. Unfortunately, SPF ferrets not infected with *H. mustelae* were not available to ascertain whether these animals also would develop MNNG-induced gastric cancer. Subsequently, aged *H. mustelae-*infected ferrets have been reported to develop gastritis, dysplasia, and gastric adenocarcinoma [66].

With respect to *H. pylori*, several *H. pylori* strains, such as the G1.1 strain [67], the TN2 strain [68], or the B128 strain [69], have the ability to colonize Mongolian gerbils, and induce gastric adenocarcinoma. This has allowed researchers to infect Mongolian gerbils with a variety strains, including their isogenic mutants, in order to investigate the importance of bacterial virulence factors in gastric carcinogenesis [67,68,70].

Interestingly, mice in general and the C57BL/6 strain in particular are proved to be remarkably resistant to colonization with various *H. pylori* strains [71,72]. Thus, alternative mouse models of gastric *Helicobacter* infection were explored. In 1990, *Helicobacter felis*, a close relative of *H. pylori* that was isolated from the cat stomach, was shown to readily colonize the mouse stomach in large numbers [73]. Several papers reported that *H. felis* had the ability to induce severe gastritis and atrophy in mice [73,74,75]. Moreover, with a longer time period of observation, analysis of *H. felis*-infected mice showed gastric metaplasia, dysplasia and invasive cancer [21,76]. After 12–16 months of infection, extensive dysplastic lesions were evident in the gastric corpus at the squamocolumnar junction (SCJ) along the lesser curvature. After more extended periods of infection (up to 2 years), large polypoid antral tumors develop and mimic closely lesions found in humans infected with *H. pylori* [77,78]. In the *H. felis* infection model, eradication of *Helicobacter* infection at early time points led to a regression of inflammation, restoration of parietal cells, reestablishment of normal architecture, and prevention against development of adenocarcinoma. Bacterial eradication at 1 year was also associated with the reappearance of parietal cells and partial restoration of architecture. Thus, eradication studies in mice have revealed that inflammation, metaplasia, and dysplasia are reversible with early eradication therapy, and that progression to dysplasia can be arrested with eradication therapy at a later time point [79,80]. In humans, eradication of *H. pylori* in patients with gastritis but not dysplasia is linked to a decrease in the incidence of gastric adenocarcinoma epidemiologically [81,82], thus supporting findings in mouse models. Antibiotic treatment to eradicate *H. pylori* in gerbils and INS-GAS mice also arrests progression to gastric lesions [73,83].

Strains of *H. pylori* that can colonize mice—the so-called “mouse adopted strains of *H. pylori*”—have been developed. Among *H. pylori* mouse adapted strains reported to date, the Sydney strain of *H. pylori* (SS1) has been the best characterized and most useful in murine model systems [20]. Relatively high levels of colonization were achieved in inbred C57BL/6 mice, while colonization levels in Balb/c, DBA/2, and C3H/He strains were lower [20]. After 8 months of infection, active gastritis and severe atrophy were observed, along with detectable levels of bacteria [20]. However, even with a 2-year follow-up, lesions did not progress to gastric cancer in mice infected with SS1 or other strains, 119p and G50, although some mice developed gastric lymphoma [84]. *H. pylori* SS1 infection did result in development of carcinomas *in situ* in C57BL/129 mice 15 months after infection [19]. *H. pylori* infection also causes gastric cancer in genetically modified mice, for example, INS-GAS mice (see below) [85].

In *H. pylori*, the *cag* pathogenicity island (cag-PAI), a 40-kb genomic fragment containing 31 genes, encodes a type IV secretion (TFSS) apparatus used to inject bacterial proteins such as the 120-kilodalton protein CagA into host epithelial cells [78]. A series of *in vitro* reports have established that injection of CagA into host cells leads to phosphorylation of CagA by host cell kinases, resulting in activation of SHP-2 tyrosine phosphatase, NF-κB signaling pathways, and MAPK signaling pathways [86,87,88]. The *H. pylori* peptidoglycan is also injected into host cells via the type IV secretory system, leading to activation of Nod1, an intracellular pathogen recognition molecule with specificity for gram-negative peptidoglycans [89], and mice deficient in Nod1 are more susceptible than wild-type (WT) mice to infection by *cag*-positive strains of *H. pylori* [89].

Interestingly, while the *H. pylori* SS1 strain was initially reported to possess an intact cag-PAI, the SS1 strain used in subsequent studies does not appear to express CagA, which may be explain to some extent the limited virulence of SS1 in mice [90]. Systemic expression of CagA in transgenic mice has led to the development of gastrointestinal and hematological malignancies [91]. mice deficient in Nod1 are more susceptible than wild-type mice to infection by *cag*-positive strains of *H. pylori* [89]. Arnold *et al.* used CagA-positive SS-1 (PMSS1, which was original strain isolated from a patient) and showed that mice infected with PMSS1 rapidly develop gastritis, gastric atrophy, epithelial hyperplasia, and metaplasia in a type IV secretion system-dependent manner [92]. These results suggest that CagA and/or the cag-PAI may play an important role in gastric carcinogenesis. Nevertheless, in mice, *cag*-negative strains such as *H. felis* appear to be at least as carcinogenic as *cag*-positive *H. pylori* strains. Although the ability of PMSS1 to inject CagA into host cells decreases gradually after 1 month infection and disappear after 3 months, strong inflammation and phenotypic changes could be sustained for more than 6 months [92]. In addition, inactivation in a *H. pylori* strain of the *cagE* gene coding for TFSS delayed the progression to carcinoma, but neoplasia ultimately developed in all infected INS-GAS mice with the *H. pylori* mutant [85]. Thus, taken together, these observations might suggest that the induction of gastric preneoplasia in mice is affected by host factors, such as the inflammatory response or other genetic factors.

Support for the importance of host genetic factors modulating gastric carcinogenesis includes observations regarding *Helicobacter* colonization of various inbred mouse strains, which revealed markedly different responses [75,76,93]. For example, the C57BL/6 strain is more sensitive than the Balb/c strain to *H. felis*-induced gastric atrophy. Potential explanations include the more T-helper-1 (Th1)-dependent immune response in C57BL/6 mice, compared to a Th2-dominant immune response in Balb/c mice [76], or possibly the reduced activity of phospholipase A2 in C57BL/6 mice [76,94]. Susceptible strains such as C57BL/6 mice show much higher levels of pro-inflammatory cytokines such as IFN-γ. The use of immunodeficient mice or mice which are deficient or overexpressing cytokines such as IFN-γ, IL-10, IL-4, or IL-7, have been useful for investigating the role of the host’s immune system in development and severity of gastritis and following mucosal changes [32,95,96,97,98,99,100,101,102]. In addition, in susceptible strains such as C57BL/6 mice, *H. felis*-induced proliferation and apoptosis are markedly increased compared with resistant strains [76]. The role of apoptosis was further studied through the combination of *Helicobacter* infection and mice which lack apoptosis-related genes, such as Fas [103,104], p53 [21], or IKK-β [105]. These studies have supported the notion that increased apoptosis is critical in the development of atrophy, metaplasia, and dysplasia.

Mouse models of *Helicobacter* infection have been used to examine the role of other co-factors in gastric carcinogenesis, such as gender, diet, and co-infection. Gender may be important, since gastric cancer is much more prevalent in men compared to women. *Helicobacter* infection of some murine strains, such as *H. felis* or *H. pylori* infection of INS-GAS mice (see below), results in greater gastric carcinogenesis in male mice compared to female mice [85,106]. However, C57BL/6 mice infected with *H. felis* did not show significant gender differences in the incidence of gastric carcinoma [21,85], suggesting the different mechanisms of carcinogenesis in these models. Indeed, some studies of *Helicobacter* infection in mice indicate that female C57BL/6 mice are more susceptible to gastric disease [107,108]. High salt diets, and diets rich in nitrates and nitrites, have been associated with an increased gastric cancer risk. Treatment with *N*-nitroso compounds, such as MNU, prior to *H. pylori* infection caused more severe preneoplastic changes and increased gastric cancer as mentioned above [109,110]. C57BL/6 mice infected with SS1 and fed a high-salt diet developed more pronounced gastric atrophy and foveolar hyperplasia [111]. Concurrent parasitic infection may also alter the effects of *Helicobacter* infection. Co-infection of C57BL/6 mice with the helminth, *Heligmosomoides polygyrus*, along with *H. felis*, reduced the severity of gastric atrophy and preneoplastic lesions seen with *H. felis* alone [112]. This was associated with a shift from the usual Th1 mucosal immune response to a polarized Th2 response. Finally, gastric atrophy due to chronic *H. pylori* infection is associated with bacterial overgrowth, which has been postulated to be an additional risk factor for gastric cancer. Germ-free INS-GAS mice infected with *H. pylori* developed less severe and delayed gastric preneoplastic lesions compared with SPF-conditioned INS-GAS mice [113], suggesting that commensal bacterial flora in the stomach also influenced the development of gastric cancer.

Mouse models of *Helicobacter*-induced gastric cancer make it possible to explore the origin of cancer cells and surrounding tissues. By using a bone marrow transplantation technique, Houghton *et al.* reported that chronic infection of C57BL/6 mice with *H. felis* results in population of the stomach with bone marrow-derived cells (BMDCs), and that these cells progress through metaplasia and dysplasia to intraepithelial cancer [114]. These findings were confirmed in an independent study, which showed that *H. pylori* infection also recruits and accumulates BMDCs in gastric epithelium [115]. Fibroblasts around gastric cancer, which have been called cancer-associated fibroblasts (CAFs) recently, are expanded by *H. felis* infection, and they were reported to be partly derived from bone marrow [31,116].

Taken together, studies to date have demonstrated that mouse models of chronic *Helicobacter* infection are robust and reproducible models that provide insights into the molecular mechanism of gastric carcinogenesis. However, there are limitations to *Helicobacter* mouse models, which include: the limited strains of *H. pylori*, particularly *cag* positive strains, that are able to colonize in mice; the slow time course for the development of tumors; the low incidence rate of advanced or invasive gastric cancer; anatomical differences (e.g., forestomach) between human and mice; the predominance of high grade dysplasia/early gastric cancers; and the absence of metastatic disease in these carcinogenesis models.

## 4. Genetically Engineered Mouse Models

Progress in genetic engineering technology leading to the development of transgenic or knockout mice has been useful for developing additional models of gastric cancer that have also shed light on the role of host genetic factors in gastric cancer development. These have included overexpression (or deficiencies) of growth factors and cytokines, as well as mutation of classical tumor suppressor genes and oncogenes. Below we have summarized six sets of animal models that have been reasonably well validated and that have provided unique insights into the pathogenesis of gastric adenocarcinoma. In addition, we mention briefly several other useful genetic models.

### 4.1. INS-GAS Mice

**Figure 2:**
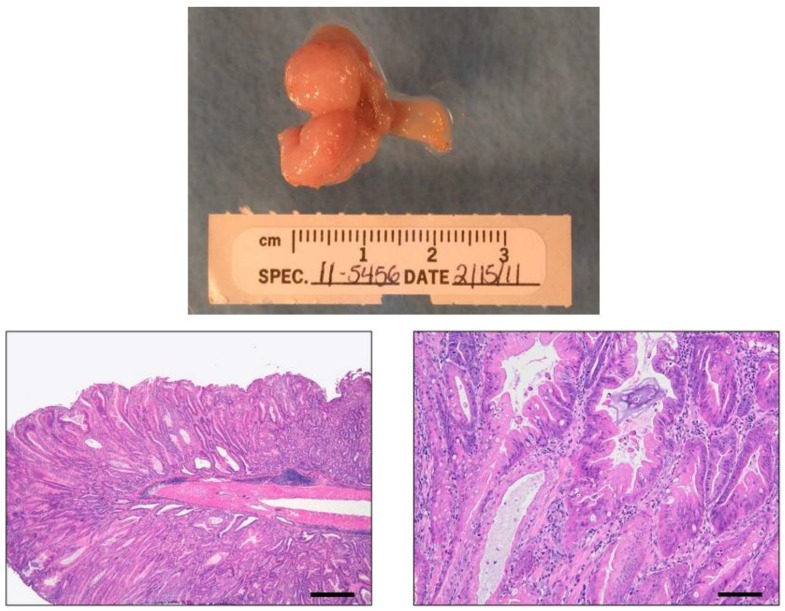
INS-GAS mouse tumor. Gross image: Stomach of a *H. pylori*-infected male INS-GAS mouse at 7 mpi showing coalescing diffuse tumors of the glandular stomach. Bottom panels: Low and high magnification H&E images of the stomach of an *Hp*-infected male INS-GAS mouse at 7 mpi showing diffuse high grade dysplastic/neoplastic glandular proliferation with lamina propria invasion consistent with the diagnosis of intramucosal carcinoma. Other features present include prominent mixed inflammation, erosions, mucosal/glandular degeneration/necrosis, glandular ectasia, oxyntic loss, severe hyperplasia, pseudopyloric and foveolar type metaplasia as well as glandular herniation.

Although Zollinger-Ellison syndrome patients associated with syndrome type I MEN are likely to develop ECL cell carcinoid tumors [117], clear evidence of epidemiological correlation between hypergastrinemia and gastric cancer in human has not been reported. However, a subset of patients infected with H. pylori show mild-moderate increases in circulating levels of amidated gastrin, and thus a role for gastrin in gastric cancer was suggested, given its known role as a growth factor for the stomach [118]. Twenty years ago, the insulin-gastrin (INS-GAS) transgenic mice were created, initially to study the role of gastrin on pancreatic islet cell formation [119]. The INS-GAS transgene consisted of the insulin promoter upstream of the human gastrin coding sequences, and resulted in the overexpression of amidated gastrin (primarily G-17) in the pancreatic β-cells, leading to two-fold elevations in serum levels of human amidated gastrin. When crossed with MT-TGF-α transgenic mice, the combination of gastrin and TGF-α was able to synergistically stimulate islet growth, although neither peptide alone was sufficient to stimulate pancreatic islet growth [120]. However, given the increases in circulating gastrin, subsequent investigations focused on the effects of gastrin on the gastric mucosa [29]. Young INS-GAS mice showed increased maximal gastric acid secretion and parietal cell mass, but interestingly progressed later to decreased parietal cell mass (atrophy), hypochloryhydria and worsening hypergastrinemia, in association with increased epithelial proliferation [29,121,122]. Over time, INS-GAS mice (in an FVB background) showed progression to gastric metaplasia and dysplasia, with the development of invasive gastric cancer in the corpus at 20 months of age (Figure 2). Infection of INS-GAS mice with *H. felis* or *H. pylori* led to accelerated development of intramucosal carcinoma (in <12 months), with submucosal invasion and intravascular invasion [29,85]. Interestingly, the FVB/N inbred strain was most susceptible, while uninfected INS-GAS mice in a C57BL/6-background also developed mild gastric corpus hyperplasia and low-grade dysplasia [123], they did not progress to gastric cancer, even in the setting of *H. felis* infection.

Although *H. pylori* infection on its own is slow to induce gastric atrophy, and is unable to induce gastric cancer in the majority of strains of WT mice, it is highly effective as a gastric carcinogen in INS-GAS mice. Thus, multiple studies from independent laboratories have now confirmed that *H. pylori* infection of INS-GAS mice leads to accelerated gastric cancer. The model has been widely used, particularly to examine the importance of various kinds of gene expressions [34,124,125,126,127,128], antagonists or inhibitors [129,130], bacterial factors or commensal flora [80,83,85,113], gender difference or sex hormones [106,131,132,133], dietary cofactors [134], and apoptotic factors [135,136] in gastric carcinogenesis, as well as to analyze non-gastric diseases such as colon cancer [137,138], intestinal crypt regeneration [139], or iron-deficiency anemia [140] (Table 1). In addition, the model has proved highly useful for studies of cancer prevention, given the high degree of reproducibility and the relative rapid development of cancer in this model.

**Table 1:** Applications of INS-GAS mouse model.

Purpose of analysis	Results
Pancreatic islet cell formation [120]	gastrin and TGF-α synergistically stimulate islet growth
*H. pylori* and gastric mucosa [29]	progression to gastric atrophy, metaplasia, dysplasia, and cancer
Colonic carcinogenesis by AOM [137,138]	progastrin, not gastrin, promotes colon carcinogenesis
Gender differences [85,106]	greater gastric carcinogenesis in male INS-GAS mice with *H. pylori*
Importance of CagE [85]	loss of cagE temporally retards but does not abrogate cancer progression
Interaction with G-Gly [124]	G-gly synergizes with amidated gastrin to stimulate acid secretion and inhibits atrophy
CCK2R and Histamine receptor inhibitors [129]	CCK2R and H2R antagonists synergistically inhibit gastric atrophy and cancer
Intestinal crypt regeneration [139]	hypergastrinemia increases regeneration of intestinal injury
Apoptosis of gastric epithelium [135,136]	gastrin induces apoptosis and contribute to gastric carcinogenesis
Gene expression profiling [125]	identify up- and down-regulating genes among 12,000 cDNA
TFF2 expression [126]	TFF2 expression in the gastric fundus was elevated in INS-GAS mice
Swedish variant of moist oral smokeless tobacco [134]	tobacco promotes cancer formation in *H. pylori*-infected INS-GAS mice
Reg-1 expression [127]	Reg1 is increased in the stomachs of *H. felis*-infected INS-GAS mice
Role of 17-beta-estradiol [131,132,133]	17beta-estradiol has protective effects on gastric cancer development
Eradication of *Helicobacter* [79,80,83]	eradication inhibits mouse gastric carcinogenesis
Commensal bacterial flora in the stomach [113]	SPF mice are more susceptible to gastric cancer than germ-free mice
Antral carcinogenesis [60,123]	gastrin suppresses antral carcinogenesis
HB-EGF, MMP-7, EMT protein [128]	neutralisation of gastrin in INS-GAS mice reduced MMP-7, HB-EGF and EMT proteins
Acetic acid and cytoreduction [130]	acetic acid could be a potent cytoreductive treatment of gastric cancer
Effect of IL-8 [34]	IL-8 promotes gastric carcinogenesis in INS-GAS mice
*H. pylori*-induced iron deficiency [140]	marked changes in expression of gastric iron transporters in *H. felis*-infected INS-GAS mice

### 4.2. Gastrin Knockout Mice

As noted above, while a subset of *H. pylori*-infected patients exhibit significant hypergastrinemia (e.g., 2-fold elevations in gastrin), most patients infected with *H. pylori* do not. In fact, many *H. pylori* infected patients with pan-gastritis who develop gastric atrophy show depressed levels of circulating gastrin. Thus, it was not entirely surprising when several laboratories reported that gastrin knockout (GAS-KO or GAS^−/−^) mice were also susceptible to stomach cancer. However, in contrast to the hypergastrinemic INS-GAS mice which developed corpus cancers, GAS^−/−^ mice exhibited antral gastric cancers. GAS^−/−^ mice (C57BL/6 strain) were first generated by Koh *et al.*, and the initial phenotype reported was fairly unremarkable, with mild changes in gastric architecture, including a slight decrease in the number of parietal and enterochromaffin-like cells [141]. However, an independent group reported that a separate line of 129/Sv GAS^−/−^ mice, showed hypochlorhydria and bacterial overgrowth, resulting in increased gastric inflammation [142]. Moreover, these GAS^−/−^ mice that were kept in conventional (non-SPF) housing conditions developed spontaneous antral tumors [143]. These findings were confirmed by an independent group, and the GAS^−/−^ mice were also found to be more susceptible to MNU-induced antral cancer compared to WT mice in the same genetic background [60].

Taken together, these reports suggest that gastrin has distinct functions in the gastric corpus and gastric antrum. Indeed, in contrast to the gastrin response observed in the corpus, hypergastrinemic INS-GAS FVB mice infected with *H. felis* showed decreased mucosal changes in the antrum relative to WT mice [123], and INS-GAS mice were found to be more resistant to MNU-induced antral tumors [60]. The antral tumor suppressive function of gastrin could be explained to some extent by its effect on stimulating acid secretion leading to inhibition of bacterial overgrowth. However, Dimaline *et al.* first reported that gastrin regulates TFF1 gene expression, providing a link between gastrin and a known tumor suppressor gene (see below). Tomita *et al.* went on to demonstrate that gastrin regulates TFF1 expression *in vivo* and *in vitro* through DNA methylation and histone modification [60]. Therefore, these studies suggest that amidated gastrin (e.g., G-17) increases corpus proliferation and cancer susceptibility, but (through TFF1) decreases antral proliferation and cancer susceptibility. The distinct effects of gastrin on proximal versus distal gastric cancers are consistent with the distinct epidemiology and behavior of tumors at these two anatomical sites, and emphasizes the importance of pathological descriptions clearly distinguishing the corpus and the antrum as separate tumor sites.

### 4.3. TFF1 Knockout Mice and Gp130 Mutant Mice

The human and mouse TFF1 (formerly known as pS2) proteins belong to the family of trefoil peptides, which are characterized by the presence of one to six cysteine-rich P domains. TFF1 proteins are normally expressed in the epithelial cells of the gastric mucosa, and are abnormally expressed in gastrointestinal diseases and various cancers. To elucidate the function of TFF1, Lefebvre *et al.* disrupted the mouse TFF1 gene by homologous recombination, generating TFF1^−/−^ mice (F2 129/Svj mixed background) [144]. Mice deficient in TFF1 expression displayed hyperplastic gastric epithelium with markedly elongated gastric pits, and multifocal intraepithelial or intramucosal carcinomas were observed in 30% of mice [144]. A recent study has shown that loss of TFF1 leads to activation of IKK complex-regulated NF-κB transcription factors, resulting in enhanced NF-κB-mediated inflammatory responses during the progression to gastric tumorigenesis [145].

A reduction in TFF1 gene expression has been observed in about 50% of human distal stomach cancers, and promoter hypermethylation has also been found rather than mutation of the TFF1 gene [146,147,148]. A well-defined positive transcriptional regulator of TFF1 is the peptide hormone gastrin [149]. Gastrin inhibits TFF1 repression and thus suppresses MNU-induced antral gastric carcinogenesis [60]. In *Helicobacter*-infected human or mouse tissues, TFF1 was moderately epigenetically repressed, but much greater TFF2 repression was observed in stomach cancer [60]. Clyne *et al.* reported that *H. pylori* bound to the TFF1 dimer *in vitro* and that this interaction enables binding to gastric mucin, suggesting that TFF1 may act as a receptor for the organism [150,151]. However, *in vivo* evidence for direct *H. pylori*-TFF1 interaction has been lacking. Thus, further studies are needed to clarify the possible association between TFF1 and *H. pylori*.

While TFF1 is expressed predominantly in foveolar surface mucous or pit cells of the stomach, TFF2 is expressed in the deeper glandular epithelium in the distal stomach and the acini of Brunners glands in the duodenum [152,153,154]. Genetically engineered mice deficient in TFF2 show a minimal phenotype, with only a slight reduction in proliferation rates in the gastric mucosa [155] but *H. pylori*-infected TFF2-deficient mice develop more advanced premalignant lesions of atrophy, metaplasia, and dysplasia than WT mice [156]. Furthermore, the fundus of gp130^F/F^*/*TFF2^−/−^ mice displayed glandular atrophy and metaplasia. These results suggest that TFF2 negatively regulates preneoplastic progression and subsequent tumor development in the stomach [157].

Gp130 is a common co-receptor for the cytokines IL-6 and IL-11. Mice with a mutation of the gp130 receptor (gp130^F/F^ mice), which abrogates Src-homology tyrosine phosphatase 2 (SHP2)-Ras-ERK signaling following gp130 engagement, have a dramatic gastric phenotype. Gp130^F/F^ mice progress rapidly to gastric neoplasia, with evidence of gastric adenomas by 3 months of age [158,159]. Interestingly, mutation of the gp130 receptor leads to downregulation of the TFF1 gene, and the phenotype of gp130^F/F^ mice in many ways mimics that of TFF1^−/−^ mice. The main cytokine driver of gp130 signaling in the stomach is IL-11, with IL-6 having little activity in the antral stomach [160,161]. IL-11 appears to promote chronic gastric inflammation and associated tumorigenesis mediated by excessive activation of STAT3 and STAT1 [162].

### 4.4. H/K-ATPase-IL-1β Transgenic Mice

**Figure 3:**
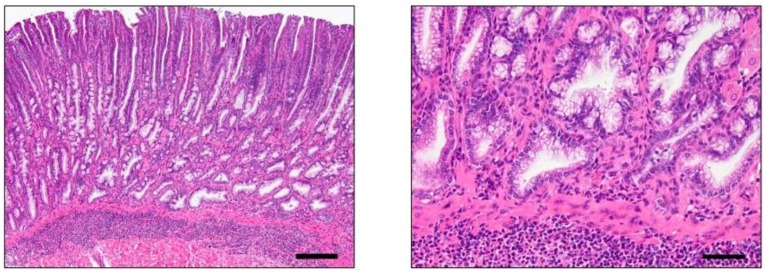
H/K-ATPase-IL-1β mouse: Inflammation, Metaplasia and Dysplasia. Low and high magnification images of the gastric corpus depicting prominent mucosal and submucosal granulocytic and lymphocytic inflammation with oxyntic loss, mucous metaplasia, foveolar and glandular hyperplasia, as well as dysplasia characterized by glandular architectural abnormalities such as misorientation, splitting, elongation, back to back formation, crowding and mild cellular atypia. (Bar: Left 100× or 160 μM, Right 400× or 40 μM).

Polymorphisms of the IL-1β that are predicted to increase IL-1β signaling have been shown to increase the risk of a number of human tumors, particularly gastric cancer [30]. IL-1β is a pleiotropic proinflammatory cytokine that has profound effects on inflammation and immunity, and is upregulated by *H. pylori* infection [30]. Tu *et al.* generated stomach-specific expression of human IL-1β in transgenic mice by using the murine H/K-ATPase promoter to direct expression of a constitutively active form of human IL-1β. H/K-ATPase-IL-1β transgenic mice (C57BL/6 background) exhibited spontaneous gastric inflammation and slow progression (over 1.5 years) to gastric atrophy, metaplasia and gastric cancer (Figure 3). In addition, infection of these mice with *H. felis* resulted in strong synergy and rapid progression (in <1 year) to cancer [163]. Thus, H/K-ATPase-IL-1β transgenic mice confirm the genetic findings in human patients that elevated expression of IL-1β represents a risk factor for gastric cancer, and that IL-1β can synergize with *Helicobacter* infection to drive cancer formation. In addition, it raises the possibility that IL-1β itself may represent a final common pathway for *Helicobacter* pathogenesis.

Several molecular mechanisms of cancer development in IL-1β transgenic mice have been suggested. First, IL-1β stimulates through the IL-1 receptor the NF-κB pathway, which is strongly associated with a variety of cancers. NF-κB activation in inflammatory cells leads to increased production of IL-6, TNF-α, and other cytokines that have been associated with cancer development. Second, IL-1β of tumor cell origin has been shown in other studies to stimulate hematological alterations manifested by extensive accumulation in the spleen of Gr-1+CD11b+ immature myeloid cells that induce tumor-mediated immune suppression [164]. Indeed, IL-1β transgenic mice showed early recruitment of myeloid-derived suppressor cells (MDSCs) to the stomach [163]. Third, a recent study suggested that IL-1β suppresses Sonic Hedgehog (Shh) gene expression in parietal cells by inhibiting acid secretion and the release of cellular calcium, followed by gastric atrophy [165].

The H/K-ATPase-IL-1β mouse model of gastric cancer has been useful for elucidating the important contributions of stromal cells in the tumor microenvironments, including CAFs or inflammatory cells. Quante *et al.* showed that at least 20% of CAFs originate from bone marrow (BM) and are derived from mesenchymal stem cells (MSCs), and that MSC-derived CAFs which are recruited to the dysplastic stomach express IL-6, Wnt5a and BMP4, show DNA hypomethylation, and promote tumor growth [31]. Moreover, CAFs are involved in creation of a niche to sustain cancer progression in SDF-1-dependent manner [33]. Tu *et al.* analyzed the role of T cells in gastric carcinogenesis by using IL-1β the transgenic model, and reported that IFN-γ overexpression suppressed gastric carcinogenesis because helper T cell (Th) 1 and Th17 immune responses were inhibited by IFN-γ through Fas induction and apoptosis in CD4 T cells [32].

Taken together, the IL-1β transgenic mouse model is considered to be one of the best mouse models of gastric cancer reported to date. In combination with other strains of genetically engineered mice, it should be useful for clarifying further the early steps of cancer initiation and the critical interactions that take place between the epithelial and stromal components of the gastric mucosa.

### 4.5. K-ras Transgenic Mice

K-ras is one of the most commonly mutated proto-oncogene in a variety of human cancers [166]. While normally its activity is tightly regulated, somatic mutations occur that render its activity constitutive and thereby oncogenic [167]. Oncogenic activations of K-ras have been found in human gastric cancers, although they are not as common (0–18%) in both intestinal type and diffuse type gastric cancers as reported in other solid tumors, such as pancreatic or colorectal cancer [168]. While oncogenic K-ras leads to increased signaling through a number of proliferative (e.g., MAPK) pathways, it has also been strongly linked to the development of chronic inflammation and cancer [169]. In genetically engineered mouse models of pancreatic cancer based on PDX1-directed K-ras mutations, significant inflammatory and stromal responses correlate with cancer progression [170]. To analyze the function of oncogenic K-ras on the stomach cancer development in mice, the K19-promoter, which targets expression to the progenitor zone of the gastric neck/isthmus [171], was used to direct expression of K-ras-V12 mutant gene. K19-K-ras-V12 transgenic mice (F2 mixed C57BL/6 × DBA background) showed an early upregulation of chemokines such as CXCL1 and recruitment of bone marrow-derived inflammatory cells and fibroblasts, following by the gradual development of parietal cell loss, metaplasia and dysplasia, in a manner that closely resembled *H. felis*-induced gastric preneoplasia and carcinogenesis [171,172]. Thus, these data suggest that K-ras-dependent chronic inflammation, leading to the recruitment of bone marrow-derived cells that contribute to the stromal microenvironment, can initiate gastric carcinogenesis.

In a separate study, investigators introduced a conditional K-ras G12D mutation in the K19-positive lineage in adult mice by crossing K19-CreERT knock-in mice with LoxP-STOP-LoxP-KrasG12D mice. The phenotype of these mice included numerous hyperplasias, metaplasias and adenomas in the stomach as well as in the oral cavity, colon and lungs [173]. Another group bred UBC9-CreERT transgenic mice with LoxP-STOP-LoxP-KrasG12D mice in order to determine the effect in mice of widespread, systemic activation of K-ras [174]. Ubiquitous K-ras activation in mice had rapid and dramatic effects on both the forestomach and glandular stomach, and resulted in severe inflammation, hyperplasia, metaplasia, and activated progenitor cells, although neoplastic changes in other organs were not detected. These latter results suggest that, amongst all the tissues in which K-ras is activated, the stomach appears to be unusually susceptible to the effects of K-ras mutation at early time points, pointing to a crucial role of K-ras activation in initiation of gastric precancerous changes.

### 4.6. Wnt1 and COX-2 Transgenic Mice

Oncogenic activations of β-catenin have been found in about 20% of intestinal type gastric cancers, but not in diffuse type gastric cancers [168,175]. On the other hand, mutation of the Apc gene, while extremely common in colorectal cancer, is rarely seen in gastric cancer [168]. Nevertheless, familial adenomatous polyposis (FAP) due to germ-line mutations in the Apc gene, and characterized by the formation of thousands of colonic polyps and a high likelihood of colon carcinoma, is also associated with an increased risk of gastric polyps and cancer [176,177]. This suggests that Wnt signaling is likely to play a causal role in gastric cancer development. Thus, mice carrying a heterozygous Apc gene mutation (Apc1648) developed gastric dysplasia and polyposis in the antrum [178]. In addition, K19-Wnt1 transgenic mice were noted to have an increase of undifferentiated gastric epithelial cells along with the spontaneous development of small preneoplastic lesions in the gastric mucosa [179]. These data support a potential role for Wnt signaling in gastric carcinogenesis.

The COX-2/PGE2 pathway is also thought to play an important role in gastric tumorigenesis. Overexpression of cyclooxygenase 2 (COX-2) is frequently detected in gastric cancer [180]. COX-2 transgenic mice, where the human COX-2 cDNA was driven from the cytomegalovirus (CMV) promoter, showed an increase in MNU-induced gastric cancer development [55]. Treatment with celecoxib, a selective COX-2 inhibitor, reduced MNNG-induced gastric cancer incidence and growth in rats [181]. A combination of sulindac (a nonspecific COX inhibitor) and antimicrobial eradication prevent progression of gastric cancer in *H. pylori*-infected INS-GAS mice [182]. Finally, hyperplastic gastritis induced by *H. pylori* is associated with upregulated COX-2 expression, and gastric hyperplasia was significantly reduced by treatment with the selective COX-2 inhibitors [183,184,185].

In 2004, K19-C2mE transgenic mice were reported that simultaneously expressed both COX-2 and microsomal prostaglandin E synthase (PGES)-1 in the gastric epithelial cells. The transgenic mice developed hyperplastic lesions, metaplasia (SPEM), and tumorous growths in the glandular stomach with heavy macrophage infiltrations [186,187]. These findings suggest that an increased level of PGE2 enhances macrophage infiltration, thus contributing to gastric tumor development. When K19-Wnt1 mice were crossed with K19-C2mE to construct compound transgenic mice (K19-Wnt1/C2mE mice), the K19-Wnt1/C2mE mice developed mucous metaplasia followed by the spontaneous development of gastric adenocarcinoma [179]. The tumors consisted of dysplastic epithelial cells, which sometimes invaded the smooth muscle layers. These results clearly indicate that the simultaneous activation of the Wnt and PGE2 pathways can promote dysplastic gastric tumors through a metaplasia-carcinoma sequence. Moreover, ablation of CD44 (a gastric cancer stem cell marker [188]) in K19-Wnt1/C2mE mice (*i.e*., CD44^−/−^K19-Wnt1/C2mE mice) suppressed gastric tumor growth [189], suggesting that CD44-targeted therapy may impair tumor growth ability.

### 4.7. Other Mouse Models of Gastric Cancer

Other murine models have been reported that show a significant gastric neoplastic phenotype, and these will be discussed briefly. These have included manipulations of genes in the TGF-β/Smad pathway, RUNX3, MLH1/MSH2, p53, KLF4 and CDH1 (Table 2).

The TGF-β/Smad signaling pathway is commonly altered in gastric cancer [190,191,192], and TGF-β1 knockout mice (mixed C57BL/6/Sv/129 background) developed severe epithelial hyperplasia and metaplasia in the stomach [193]. Hahm *et al.* established TGF-β2 dominant-negative mice (mixed C57BL/6 × Sv/129 background) under the TFF1 promoter, and these mice showed a higher proliferation index and a higher incidence of gastric cancer with *H. pylori* infection [194]. Heterozygous Smad4 knockout mice (mixed C57BL/6 × Sv/129 background and C57BL/6 background) exhibited spontaneous gastric tumor development [195,196]. Interestingly, T-cell specific deletion of Smad4 induces gastric tumors, as well as colon, duodenal and oral cavity tumors, with induction of inflammatory cytokines [197,198].

**Table 2:** Mouse models of gastric cancer.

Model	Incidence	Duration	Location	Phenotype
MNU	<70%	12 months	Antrum	AdenoCa, Dysplasia [11,47,48,49]
*H. felis*	80%	18 months	SCJ/Transition	AdenoCa, Dysplasia, Metaplasia, Atrophy [21]
MNU + *H. pylori*	80%	12 months	Antrum	AdenoCa, Dysplasia, Metaplasia, Atrophy [110]
MNU + *H. felis*	100%	36 weeks	Antrum	AdenoCa, Dysplasia, Metaplasia, Atrophy [60]
CEA/SV40	100%	50 days	Antrum	AdenoCa, Dysplasia, Invasion to submucosa and duodenum [24]
MMTV/Ad12	82%(male)	3–4 months	SCJ	AdenoCa, AdenosquamousCa [26]
HPV-16	100%	246–352 days	Antrum	Carcinoid, Metastasis to lymph node and liver [27]
MTH1^−/−^	13%	18 months	Antrum	AdenoCa, Dysplasia, Hyperplasia, Lung and liver tumors [199]
TFF1^−/−^	30%	5 months	Antrum	Intramucosal carcinoma, Hyperplasia, Activation of NF-kB [144,145]
Smad4^+/−^	100%	12–18 months	Corpus/Antrum	AdenoCa, Dysplasia, Hyperplasia, Duodenal tumor [195,196]
GB-Smad4^F/F^	100%	12–18 months	Antrum	Dysplasia, Hyperplasia [197]
INS-GAS	75%	20 months	Corpus	AdenoCa, Dysplasia, Metaplasia, Atrophy, Synergized with *H. felis* [29]
GAS^−/−^	60%	12 months	Antrum	Dysplasia, Metaplasia, Atrophy, Susceptible to MNU [60,143]
Gp130^F/F^	100%	6 months	Antrum	Adenoma, Decreased TFF1 expression [158,159]
IL-1β	<70%	12 months	Transition	AdenoCa, Dysplasia, Metaplasia, Atrophy, Synergized with *H. felis* [163]
K19/K-ras	37.5%	16 months	Corpus	Dysplasia, Metaplasia, Atrophy [172]
Wnt1/C2me	100%	20 weeks	SCJ	AdenoCa, Dysplasia, Metaplasia, Attenuated by CD44 ablation [179,189]
CDH1^+/−^ + MNU	45.8%	40 weeks	Antrum	Signet-ring cell Ca, Adenoma [56]
CDH1/p53	100%	12 months	Corpus	Poorly differentiated AdenoCa, Signet-ring cell Ca [200]
RUNX3^−/−^ + MNU	71%	52 weeks	Corpus/Antrum	AdenoCa, Metaplasia, Hyperplasia [201]
Villin-KLF4^F/F^	29%	80 weeks	Antrum	Adenoma, Susceptible to MNU [57]

RUNX3 is a member of the RUNX gene family which regulates the Smad gene family transcription and TGF-β signaling. RUNX3 is frequently inactivated in gastric cancer by protein mislocalization [202]. RUNX3 knockout mice (F2 offspring) showed elongated gastric glands and increased proliferation in the gastric mucosa [203]. Ito *et al.* recently reported that RUNX3 knockout mice (Balb/c background) showed loss of chief cells and development of SPEM, and also displayed higher susceptibility to adenocarcinoma by treatment with MNU [201], supporting a role for RUNX3 as a tumor suppressor of gastric cancer.

Interestingly, alterations in p53 and DNA mismatch repair genes in mice have not produced dramatic gastric phenotypes, pointing to important differences between mice and humans. Alterations in DNA mismatch repair genes, such as MLH1 and MSH2, are associated with the Lynch syndrome (formerly known as hereditary non-polyposis colorectal cancer or HNPCC), which is characterized with increases in not only colorectal cancer but also ovarian, endometrium, liver, skin, brain, and gastric cancer [204]. However, mice lacking MLH1 or MSH2 do not develop gastric cancer, even with *H. felis* infection (Fox JG and Wang TC, unpublished data). Similarly, the p53 gene is the most commonly mutated tumor suppressor in a wide variety of human cancers. However, while *H. felis*-infected p53 hemizygous mice were reported to have a higher proliferative index and a higher gene mutation frequency than the infected control mice [205,206], they did not show increased progress to gastric cancer, although p53 heterozygous mice were more sensitive than WT counterparts to MNU [50,207]. However, a subsequent study demonstrated that the incidence of pre-neoplastic and invasive gastric carcinomas was decreased in p53 hemizygous mice [21], and an independent group reported that no differences in gastric apoptotic or proliferation indices between p53^+/+^ and p53^+/–^ mice after infection with *H. pylori* SS1 strain [208]. These results point to the limitations of using constitutive p53 knockout mice in modeling gastric cancer in mice, given the likely distinct roles of p53 in epithelial cells and inflammatory cells.

Krüppel-like factor 4 (KLF4) is a potential tumor suppressor in patients with various cancers, including gastric cancer [209]. Disruption of Klf4 in mice using the Villin-Cre-mediated system, which targets not only the intestine but also antral stem/progenitor cells [57] induced spontaneous antral tumors. MNU treatment enhanced cancer development in these mice. Therefore, inactivation of Klf4 in Villin-positive gastric progenitor cells can lead to transformation of the gastric mucosa and tumorigenesis of the gastric antrum.

To date, most murine models that develop gastric cancer have shown similarities to the well differentiated, intestinal-type of gastric cancer, but not to the diffuse type of gastric cancer. In human gastric cancer, loss of expression of the CDH1 gene encoding for E-cadherin has frequently been detected [210], often due to promoter hypermethylation [168,211], particularly in diffuse-type lesions. Germline mutations of the CDH1 gene have been observed in hereditary diffuse type gastric cancer [212]. CDH1 knockout mice (C57BL/6 background) have been generated, and in the MNU carcinogenesis model, CDH1^+/−^ mice developed signet ring cell carcinoma with a high tumor incidence rate [56]. Recently Shimada *et al.* generated conditional CDH1 and p53 double knockout mice under targeting by the H/K-ATPase promoter [200]. In these conditional double knockout mice, intramucosal and invasive cancers composed of signet ring cells were found from 6 to 9 months, while mice lacking only the CDH1 gene developed no cancers. These observations support the notion that CDH1 plays a critical role in especially diffuse type gastric cancer, but mouse gastric cancer development requires not only CDH1 loss but also an additional mutation or carcinogen exposure.

## 5. Models of Precancerous Change

*H. pylori-*associated gastric cancer in humans is preceded by a cascade of precancerous lesions, and cancer emerges following a number of discrete stages, including chronic gastritis, gastric atrophy, intestinal metaplasia, and dysplasia. Thus, in addition to mouse models of gastric cancer, there are a number of genetically engineered models that show decreased numbers of parietal cells, or gastric atrophy, along with metaplasia. In this section, we review briefly a number of models of atrophy and metaplasia that could be considered for use in experimental studies (Table 3). However, most of these models do not appear to progress to neoplasia, and most have not been examined for susceptibility to cancer in response to carcinogens.

**Table 3:** Mouse models of precancerous changes.

Model	Duration	Phenotype
*H.pylori* (SS-1)	6–9 months	Atrophy, SPEM [20]
TGF-α transgenic	3 months	Atrophy [122,213]
H/K-ATPase/DT	28–80 days	Atrophy [214]
H/K-ATPase/Tk	Ganciclovir treatment	Atrophy [215]
H/K-ATPase-α^−/−^	10 weeks	Atrophy [216]
H/K-ATPase-β^−/−^	17 days	Atrophy [217,218]
NHE2^−/−^	17 days	Atrophy [219]
Car9^−/−^	4 weeks	Atrophy [220]
CCK2R^−/−^	18 weeks	Atrophy [221,222]
H/K-ATPase/Shh^−/−^	3–8 months	Pit cell hyperplasia, loss of parietal cell function [223]
DMP-777	7–14 days	Atrophy, SPEM [224,225]
L-635	7 days	Atrophy, SPEM [226]
Cdx2 transgenic	120 days	Intestinal metaplasia [227,228]
Cdx1 transgenic	120 days	Intestinal metaplasia [229]

### 5.1. Models of Gastric Atrophy

There are a number of genetically engineered mouse models, which exhibit parietal cell loss, *i.e*., gastric atrophy. Indeed, one strategy to achieve more rapid progression to gastric cancer is to ablate parietal cells, which appear to protect the homeostasis of the stomach in part through acid secretion. As mentioned above, MT-TGF-α transgenic mice show a form of atrophic gastritis, with loss of parietal cells along with foveolar hyperplasia [122,213]. Using the promoter of the β-subunit of the H/K-ATPase gene driving diphtheria toxin or herpes simplex 1 thymidine kinase, researchers were able to partially ablate parietal cells [214,215]. Interestingly, with loss of mature parietal cells in both of these models, there was concomitant loss of chief or zymogenic cells, along with an increase in progenitor cells. Thus, accumulating data indicate that the parietal cell itself or its secreted products plays a critical role on maintaining gastric gland homeostasis and controlling the development of cancerous changes. 

In many of these mouse models with impaired parietal cell function, hypochlorhydria leads to hypergastrinemia, which then induces foveolar hyperplasia. Thus, mice with a disrupted H/K-ATPase β-subunit gene exhibit abnormal parietal cell morphology, achlorhydria, hypergastrinemia, and hypertrophied gastric mucosa [216,217]. When crossed with GAS^−/−^ mice, gastric hypertrophy in the H/K-ATPase-β-deficient mice disappeared, confirming that this phenotype is largely hypergastrinemia-dependent [218]. Similarly, targeted disruption of the Na+/H+ exchanger isoform 2 (NHE2) gene or carbonic anhydrase gene have also been reported to induce parietal cell loss [219,220]. Transgenic mice with parietal cell-specific deletion of Sonic Hedgehog (Shh) (H/K-ATPase-Cre/Shh^−/−^) also developed gastric hypochlorhydria, hypergastrinemia, and a phenotype that resembled foveolar hyperplasia with hyperproliferation of surface mucous cells [223]. Therefore, Shh may also function as a gastric morphogen, regulating gastric epithelial cell function and differentiation. In contrast, mice deficient in gastrin or its receptor cholecystokinin-B exhibit impaired acid secretion and reduced parietal cell numbers, but do not show foveolar hyperplasia [141,221,222,230]. As noted above, gastrin knockout mice are more susceptible to mouse induced antral carcinogenesis, but most other mouse models have not been tested in carcinogenesis trials.

### 5.2. Models of Metaplasia

Recently, it has become clear that there are two distinct types of metaplasia in the stomach that precede and are associated with gastric cancer [231]. (1) Spasmolytic polypeptide-expressing metaplasia (SPEM), also known as pseudopyloric metaplasia, is characterized by the presence of TFF2- and MUC6-immunoreactive cells in the gastric fundus with morphological characteristics similar to deep antral gland cells or Brunner’s gland cells. (2) The other is classical intestinal metaplasia (IM), which is characterized by the presence of cells with the morphology of goblet cells and expression of MUC2 and TFF3. Both types of metaplasia are Alcian blue-positive. For many years, IM was thought to be a direct precursor of gastric cancer; however, while the association of intestinal-type cancers with chronic *H. pylori* infection and oxyntic atrophy in human is well accepted, little evidence directly links intestinal metaplasia with dysplastic transformation [232]. Indeed, many investigators have come to the conclusion that SPEM is more likely to be the relevant precursor to gastric adenocarcinoma [233].

In most of the mouse models progressing to dysplasia described above, including mouse models of *H. felis* infection and transgenic mouse models (such as INS-GAS and H/K-ATPase-IL-1β mice), which demonstrate severe parietal cell loss, the fundic mucosa is replaced with a mucous cell metaplasia which was shown to be TFF2-expressing SPEM. Importantly, in these mouse models which progress to cancer, SPEM represents the only observed metaplasia, with no goblet cell IM being present. In addition, a short term model of parietal cell ablation has been described, using the neutrophil elastase inhibitor, DMP-777, which was shown to be a parietal cell-specific protonophore. DMP-777 treatment has allowed the examination of SPEM induction after acute oxyntic atrophy in the absence of significant inflammatory infiltrate. Mice or rats treated for 3 days with DMP-777 demonstrate a rapid loss of parietal cells [224,225]. The acute oxyntic atrophy is followed by SPEM in the fundus after 7–10 days. A structurally related β-lactam compound L-635, which retains potent parietal cell protonophore activity, but does not have any significant activity against neutrophil elastase, also induces gastric atrophy. In contrast with DMP-777 treatment, submucosal and intramucosal inflammatory infiltration was observed, and L-635 caused a more rapid and marked mucous cell TFF2-positive metaplasia [226]. These results indicated that a combination of parietal cell loss and inflammation could potentiate the development of SPEM.

Transgenic mice expressing the intestine-specific homeobox gene, Cdx2, under H/K-ATPase or Foxa3 promoter showed the disappearance of parietal cells and the replacement by classical IM [227,228]. Moreover, mice overexpressing Cdx1, another intestine-specific homeobox gene, also developed IM [229]. However, no further progression to gastric carcinogenesis was observed in these transgenic mice, consistent with the notion that IM may not be the primary precursor of gastric cancer. However, the effect of *Helicobacter* infection on the gastric phenotype of these mice has not been assessed. Thus, further studies are needed to clarify the precise relationship between classical intestinal metaplasia and malignant transformation.

## 6. Conclusions and Future Perspectives

Numerous mouse models with interesting gastric phenotypes are now available for studies of gastric carcinogenesis. These include transgenic mice (e.g., INS-GAS, H/K-ATPase-IL-1β, K19-Wnt1/C2mE), knockout mice (GAS^−/−^, Tff1^−/−^, gp130^F/F^), *Helicobacter* infection (*H. felis*, *H. pylori*) and carcinogen (MNU) models. These models have defined potential roles for gender, diet, bacterial flora, inflammatory cytokines, T helper immune response, acid secretion, virulence, and colonization properties of *H. pylori* strains and host genetic background. Reasonable models are now available for studies of early stage pathogenesis and cancer prevention.

The mouse stomach consists of four different parts: forestomach, cardia, corpus, and antrum. Some models have been described in which forestomach tumor, *i.e*., squamous cell carcinoma, is induced with a carcinogen or by genetic alterations [26,46,47]. These models are not useful for investigating the mechanism of human gastric cancer, as humans do not have a forestomach and gastric squamous cell carcinoma is very rare. While there has been no model that resembles human cardia cancer, a variety of corpus and antral cancer models have been reported as described above. However, in order to select appropriate mouse models of gastric cancer, investigators need to consider the histopathology, the cell type of origin and the geographic distribution of the resultant cancer. Sometimes, a given physiological stimuli will show a different phenotype between the corpus and antrum, such as seen in gastrin transgenic and knockout mice [29,60,143].

Unfortunately, genetic models of metastatic gastric cancer similar to those developed for pancreatic cancer, comprising two or three mutations targeted to specific cell lineages, are not available. These models could facilitate preclinical studies and the testing of newer therapeutics. The major limitations in the development of these models have been the weak and scattered activity of promoters used in the stomach and the lack of stomach specific promoters that target antral progenitors but are not expressed elsewhere (Table 4). K19-Cre and Foxa3-Cre constructs, for example, are expressed in the stomach and can be used as a model for the analysis of gastric cancer, but these promoter constructs are also expressed in the intestine, colon and pancreas, as well as other tissues [105,172]. Recent lineage tracing studies revealed several stem/progenitor markers of gastrointestinal tissue, including Lgr5, Bmi1, Hopx, Lrig, Sox2, and Sox9 [234,235,236,237,238,239]. Among them, Lgr5, Lrig, and Sox2 have been reported to be expressed in the mouse stomach. Lgr5 positive cells are likely to be stem cells in the gastric antrum [234,240], and can be used for mutagenic targeting to the distal stomach, but Lgr5 is also widely expressed in the intestine and elsewhere making targeted stomach mutations impractical. Similarly, Lrig and Sox2 are expressed in the intestine and other organs as well as the stomach. Villin-Cre constructs also can target antral glands, but are also expressed in whole intestine [241]. On the other hand, stomach specific promoters such as the H/K-ATPase promoter are not ideal since they target more mature parietal cells, which are unlikely to be the precursor lineage of distal gastric cancers.

**Table 4:** Promoters for establishing gene expression in the stomach.

Gene	Location	Lineage tracing in the stomach
TFF1	Surface of stomach (pit cell area)	
TFF2	Isthmus of corpus & base of antrum	Give rise to parietal, mucous neck, and chief cells [242]
H/K-ATPase	Corpus (parietal cell)	Give rise to all gastric lineages of the corpus glands with Notch activation [243]
Foxa3	Whole stomach, other organ from endoderm	
K19	Whole stomach, intestine, colon, *etc*.	
Lgr5	Cardia, Antrum, intestine, colon, *etc.*	Give rise to all major cell types in the cardia, antrum and transition zone [240]
Sox2	Corpus, Antrum, Esophagus, Forestomach, *etc*.	Give rise to all major cell types in the corpus and the antrum [236]
Mist1	Corpus (chief cell), Brunner gland, pancreas	Give rise to chief cell and drug-induced SPEM [226]
Villin	Antrum, intestine, colon	Give rise to all gastric lineages of the antral glands with IFN-γ treatment [241]

One potential candidate for a gastric targeting vector might be TFF1, which shows specific expression in foveolar cells of the stomach. To date, one study has reported the use of the TFF1 promoter to disrupt TGF-β2 specifically in the stomach [194]. Further studies using TFF1-specific gene engineering should be considered. Both TFF2-CreERT and Mist1-Cre mice are available. In the corpus, TFF2 and Mist1 are known to be potent progenitor cell lineages. Mist1-expressing cells in the stomach give rise to SPEM after stimulation [226]. TFF2mRNA-expressing cells are localized to the isthmus and are progenitors for mucus neck, parietal and zymogenic cells, but not for pit or enterochromaffin-like cell lineages [242]. TFF2 is also expressed in the base of the antral glands, suggesting that TFF2 could be a potent candidate promoter vector for targeting the stomach. In order to obtain stomach-specific gene control, other stomach-specific stem/progenitor markers should be explored by using emerging lineage tracing technique *in vivo* and stem cell culture methods *in vitro*.

Recent exome sequencing or GWAS studies have clarified significant gene mutations in human gastric cancer, such as PSCA, PLCE1, FAT4 and ARID1A [244,245,246,247]. Newer mouse models which contain these gene alterations should be useful for investigating the function of these genes. In turn, comprehensive analysis of genetic and epigenetic changes of mouse gastric cancer should also be helpful for identifying additional genetic changes or epigenetic modifications which are not covered by exome sequencing or GWAS studies focusing on human gastric cancer.

In summary, several mouse models of gastric cancer are available, which have distinct mechanisms, as well as different tumor phenotypes such as time course, locations, and pathology. Researchers are thus able to use appropriate mouse models for their studies and are commensurate with their rationale and hypothesis. Newly emerging research methods, including lineage tracing or genome-wide comprehensive analysis, and also by examining non-epithelial targets, should prove helpful for understanding both the cause and ultimately the cure of gastric cancer.
